# The ESEP study: Salpingostomy versus salpingectomy for tubal ectopic pregnancy; The impact on future fertility: A randomised controlled trial

**DOI:** 10.1186/1472-6874-8-11

**Published:** 2008-06-26

**Authors:** Femke Mol, Annika Strandell, Davor Jurkovic, Tamer Yalcinkaya, Harold R Verhoeve, Carolien AM Koks, Paul JQ van der Linden, Giuseppe CM Graziosi, Andreas L Thurkow, Annemieke Hoek, Lars Hogström, Ingemar Klinte, Kerstin Nilsson, Norah M van Mello, Willem M Ankum, Fulco van der Veen, Ben WM Mol, Petra J Hajenius, the European Surgery in Ectopic Pregnancy (ESEP) study group

**Affiliations:** 1 Department of Obstetrics and Gynaecology, Academic Medical Centre, University of Amsterdam, Amsterdam, The Netherlands; 2 Department of Obstetrics and Gynaecology, Sahlgrenska University Hospital, Göteborg, Sweden; 3 King's Early Pregnancy Unit, King's College Hospital, London, UK; 4 Department of Obstetrics and Gynaecology, Wake Forest University School of Medicine, Winston-Salem, North Carolina, USA; 5 Department of Obstetrics and Gynaecology, Onze Lieve Vrouwe Gasthuis, Amsterdam, The Netherlands; 6 Department of Obstetrics and Gynaecology, Maxima Medical Centre, Veldhoven, The Netherlands; 7 Department of Obstetrics and Gynaecology, Deventer Hospital, Deventer, The Netherlands; 8 Department of Obstetrics and Gynaecology, Antonius Hospital, Nieuwegein, The Netherlands; 9 Department of Obstetrics and Gynaecology, Sint Lucas Andreas Hospital, Amsterdam, The Netherlands; 10 Department of Obstetrics and Gynaecology, University Medical Centre Groningen, Groningen, The Netherlands; 11 Department of Obstetrics and Gynaecology Kärnssjukhuset Skövde, Sweden; 12 Department of Obstetrics and Gynaecology Norra Älvsborgs Läns Sjukhus (NÄL) Trollhättan, Sweden; 13 Department of Obstetrics and Gynaecology Kvinnokliniken, Universitetssjukhuset Örebro, Sweden; 14 Centre for Reproductive Medicine, Academic Medical Centre, University of Amsterdam, Amsterdam, The Netherlands

## Abstract

**Background:**

For most tubal ectopic pregnancies (EP) surgery is the treatment of first choice. Whether surgical treatment should be performed conservatively (salpingostomy) or radically (salpingectomy) in women wishing to preserve their reproductive capacity, is subject to debate. Salpingostomy preserves the tube, but bears the risks of both persistent trophoblast and repeat ipsilateral tubal EP. Salpingectomy, avoids these risks, but leaves only one tube for reproductive capacity. This study aims to reveal the trade-off between both surgical options: whether the potential advantage of salpingostomy, i.e. a better fertility prognosis as compared to salpingectomy, outweighs the potential disadvantages, i.e. persistent trophoblast and an increased risk for a repeat EP.

**Methods/Design:**

International multi centre randomised controlled trial comparing salpingostomy versus salpingectomy in women with a tubal EP without contra lateral tubal pathology. Hemodynamically stable women with a presumptive diagnosis of tubal EP, scheduled for surgery, are eligible for inclusion. Patients pregnant after in vitro fertilisation (IVF) and/or known documented tubal pathology are excluded. At surgery, a tubal EP must be confirmed. Only women with a tubal EP amenable to both interventions and a healthy contra lateral tube are included. Salpingostomy and salpingectomy are performed according to standard procedures of participating hospitals. Up to 36 months after surgery, women will be contacted to assess their fertility status at six months intervals starting form the day of the operation.

The primary outcome measure is the occurrence of spontaneous viable intra uterine pregnancy. Secondary outcome measures are persistent trophoblast, repeat EP, all pregnancies including those resulting from IVF and financial costs. The analysis will be performed according to the intention to treat principle. A cost-effectiveness analysis will be performed within a decision analysis framework, based on costs per live birth, including IVF treatment whenever a spontaneous pregnancy does not occur. Patients' preferences will be assessed using a discrete choice experiment.

**Discussion:**

This trial will provide evidence on the trade off between salpingostomy and salpingectomy for tubal EP in view of the pros and cons of both interventions and will offer guidance to clinicians in making the right treatment choice.

**Trial registration:**

Current Controlled Trials ISRCTN37002267

## Background

In the treatment of tubal ectopic pregnancy (EP), laparoscopic surgery remains the cornerstone of treatment [1]. In the absence of randomised data, the question as to whether surgical treatment should be performed either conservatively (salpingostomy) or radically (salpingectomy) in women with desire for future pregnancy is subject to ongoing debate.

Since the first study demonstrated the potential effectiveness of salpingostomy, this treatment has been compared with salpingectomy in numerous non-randomised studies [2]. Pooled data showed no beneficial effect of salpingostomy on intra uterine pregnancy (IUP) whereas there is an increased risk of repeat EP [3,4]. Based on these findings, the Royal College of Obstetricians and Gynaecologists guideline advises salpingectomy as the preferred standard surgical approach for tubal EP [5]. However, there are good reasons to question this advice. Interpretation of the pooled data is troublesome since many of the original studies failed to report essential details, e.g. time to pregnancy, presence of the desire for future pregnancy and whether subsequent pregnancies occurred either spontaneously or after fertility treatment, such as in vitro fertilization (IVF). Only a few non-randomised studies have taken these matters into account and came to different conclusions [6-11]; The IUP rates were higher and the time to an IUP was shorter after salpingostomy compared to salpingectomy. Especially in women with a history of bilateral tubal pathology, salpingostomy offered better IUP rates than salpingectomy, albeit at the cost of an increased risk for repeat EP [6-8,10]. In women without a history of tubal pathology this benefit was less clear and also in these women there was an increased risk for repeat EP [8]. In view of these data, we feel that the most effective type of surgery for women with a tubal EP in the presence of contra lateral tubal pathology with desire for future pregnancy is salpingostomy. In women without contra lateral tubal pathology, the most optimal surgical treatment is currently unknown.

### Objective

To study whether the potential advantage of salpingostomy, i.e. a better fertility prognosis as compared to salpingectomy, outweighs the potential disadvantages of this treatment, i.e. persistent trophoblast and an increased risk for repeat EP in women with a tubal EP without contra lateral tubal pathology.

## Methods

### Participating centres

This study is an international multi centre randomised controlled trial, originally in a Dutch-Swedish-British collaboration since October 1^st^, 2005. During the study period, other centres were contacted to participate. Since June 1^st^, 2006, a centre in North Carolina (USA) has joined the collaboration.

### Inclusion criteria

Hemodynamically stable women ≥ 18 years of age with a presumptive diagnosis of tubal EP who are scheduled for surgery, are eligible for the trial. Excluded are women without desire for future pregnancy, patients pregnant after IVF, patients with a pregnancy in a solitary tube and those patients with a contra lateral tubal occlusion or hydrosalpinx as documented earlier at hysterosalpingography or laparoscopy or as found during surgery for the index EP.

### Ethical considerations

Approval for this study was obtained from the Medical Ethical Committees of the Academic Medical Centre, Amsterdam, The Netherlands, Sahlgrenska University Hospital, Göteborg, Sweden, Kings Hospital, London, UK, and Wake Forest University School of Medicine, Winston-Salem, North Carolina, USA.A quality assessment has been made and approved by three external referees, experts from the field by the Netherlands Organization for Health Research and Development (ZonMw).

In each patient fulfilling the inclusion criteria, written informed consent is obtained before randomisation. Women refusing participation are registered.

### Randomisation

Randomisation is performed during surgery by accessing a central internet-based randomisation program. Randomisation is stratified for hospital, patient's age and history of tubal pathology (i.e., previous EP, previous tubal surgery, and previous pelvic inflammatory disease).

### Interventions

At surgery, which can either be performed laparoscopically or by laparotomy, the presence of a tubal EP must be confirmed. Patients with tubal rupture will be excluded, whenever this interferes with the possibility to perform salpingostomy. The surgeon will assess the status of the contra lateral tube during the procedure. If, according to the surgeon, the condition of the contra lateral tube renders future pregnancy unlikely in case the patient will be randomised to salpingectomy for the index tubal pregnancy (i.e., hydrosalpinx, severe peritubal adhesions, malformations, or other reasons), the patient is excluded. Thus, only patients with a tubal EP that allows both interventions, and a contra lateral tube that would allow spontaneous conception in case of salpingectomy, are being included in the study.

Whenever necessary, laparoscopy may be converted to open surgery. Salpingostomy is performed following local procedural standards used in the participating hospitals. Preferably linear salpingostomy is performed, but other methods are allowed. Whenever necessary, salpingostomy may be converted to salpingectomy, e.g. in case of uncontrollable bleeding. A complete salpingectomy is performed following local procedural standards of the participating hospitals. All methods of treatment are registered in the Case Record Form.

### Follow-up

#### Short term follow-up

Complications during the immediate postoperative period are registered in the Case Record Form. To identify persistent trophoblast in both treatment groups, serum hCG is measured postoperatively on a weekly basis until undetectable in both treatment arms to identify persistent trophoblast. Serum hCG concentrations are expressed in IU/L (conversion factor to SI unit, 1.00 according to the World Health Organization Third International Standard 75/537). Persistent trophoblast is defined as post operative rising or plateauing serum hCG concentrations [12].

#### Long term follow-up

**Figure 1:**
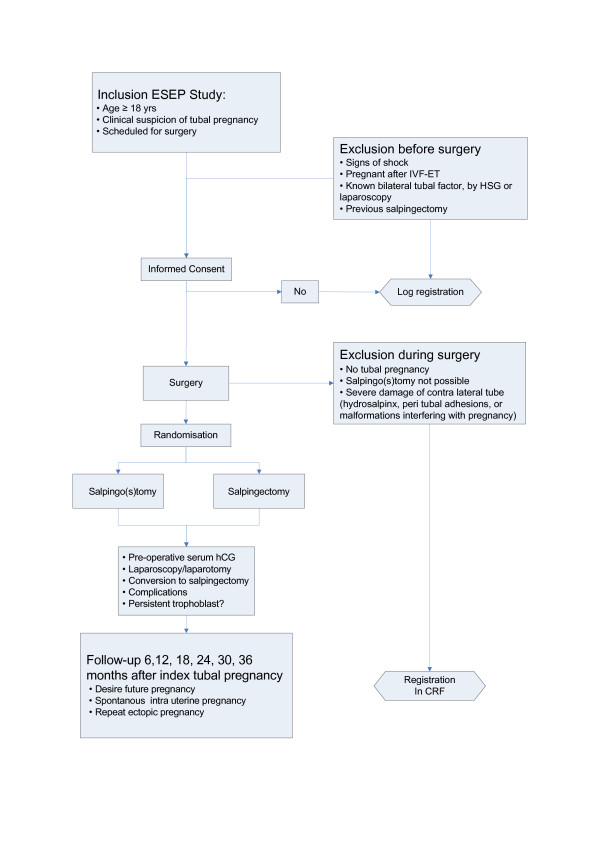
Flowchart ESEP study.

To assess fertility after the operation of the index tubal EP, the patients are contacted by means of a questionnaire, every six months for a period of 36 months. The questionnaire focuses on the presence of a desire for pregnancy, unprotected sexual intercourse with a chance of spontaneous conception, contraceptive use, infertility treatment, and the occurrence of any pregnancies and their outcomes (Figure 1).

### Outcome measures

#### Primary outcome measure

The primary outcome measure is the time to the occurrence of a spontaneous viable IUP. A viable IUP is defined as a pregnancy visible at ultrasound at a gestational age of ≥ 12 weeks with fetal cardiac activity, or the delivery of a child. If an IUP does not occur, follow-up ends on the day of the last consultation.

#### Secondary outcome measures

Secondary outcome measures are persistent trophoblast, repeat EP, all pregnancies including those resulting from IVF and financial costs. Patients' preferences will also be assessed.

Persistent trophoblast is defined as rising or plateauing serum hCG concentrations postoperatively and is primarily treated with systemic methotrexate (MTX) or otherwise if necessary [12].

Repeat EP is defined as a visible EP at ultrasound, a pregnancy of unknown location (PUL) with a serum hCG above the discriminatory zone or as a persisting PUL for which surgical or medical treatment with MTX is installed. Failing PULs, which are managed expectantly with an uneventful decline of serum hCG to an undetectable level, will be reported separately and are not considered repeat EPs. The date of occurrence of an EP or failing PUL will also be determined from the first day of the last menstrual period.

Costs are expressed as direct costs, for which data on both costs and used resources are assessed in a subset of the participating hospitals.

Patients' preferences are assessed by an online questionnaire using a discrete choice experiment (DCE) based on characteristics of both interventions and will be compared with a control group, recruited among women visiting the infertility clinics in a subset of the participating hospitals.

### Statistical analysis

The analysis is performed according to the *intention to treat *principle. Short term outcome measures are expressed in RR and their 95% confidence intervals.

Future fertility is assessed by means of life table analysis. Kaplan-Meier curves are constructed, estimating the cumulative probability of spontaneous IUP and repeat EP over time. The assessment of fertility status is censored for those periods when women used contraceptives or did not have sexual intercourse. In case a spontaneous viable IUP does not occur, follow up ends at the last date of consultation, or at the moment when either IVF or tubal surgery is performed. Spontaneous conceptions that occur after failed IVF treatment will be registered, but these pregnancies will not be considered as endpoint in the analysis. The log-rank test is used to test differences between the Kaplan-Meier curves for statistical significance. The differences between both treatment modalities are expressed as a FRR with 95% confidence interval, calculated through Cox proportional hazard analysis.

A cost-effectiveness analysis will be performed within a decision analysis framework, based on outcome of costs per live birth, including IVF programs in case a spontaneous pregnancy does not occur.

Patient's preferences will be analysed by differences in outcome of the DCE.

### Sample size

The IUP rate after salpingectomy is assumed to be 40% after 36 months and the median time to pregnancy in this group is 1.4 year [8]. We consider an increase of the IUP rate by 10–15% after salpingostomy compared to salpingectomy clinically relevant to overcome the disadvantages of persistent trophoblast and repeat EP. In order to prove a reduction of the median time to pregnancy from 1.4 year to 1 year, 225 patients in each group are required (significance level of 5%, a power of 80%, and 5% loss to follow up in both groups, 2-sided test).

### Interim analysis

An interim analysis will be performed after the inclusion of 150 women. This analysis will be done by an independent Data and Safety Monitoring Committee (DSMC) that will not be aware of the allocation of treatment. The statistical analysis will be performed according to O'Brien Fleming's rule. The decision to unblind treatment allocation is at the discretion of the DSMC. The DSMC provides a recommendation in a report to the trial coordinators. The decision to terminate or continue the study will be made in consultation with the participating centres.

### Subgroup analyses

Pre conceived subgroup analyses are planned for age (under and over 30 years), history of a previous EP, pre-operative serum hCG-level (< 3,000 IU/l, 3,000–6,000 IU/l, and > 6,000 IU/l), and size of the ectopic mass (less or more than 4 cm).

## Discussion

In industrialized countries the incidence of EP is approximately 1 to 2% of all pregnancies [13-15]. Apart from the immediate treatment burden and the major psychological impact of an early pregnancy loss, there is also concern about the effect on future fertility.

To date, there are no randomised controlled trials, which have examined the potential benefits of performing salpingostomy in women with no evidence of contra lateral tubal damage as compared to salpingectomy. Despite this lack of evidence, clinicians prefer a salpingectomy over a salpingostomy in the presence of a healthy contra lateral tube [16]. This preference is based on the small risk of tubal bleeding in the immediate postoperative period, the potential need for further treatment for persistent trophoblast and the possibility of a repeat EP in the conserved tube. Moreover, many clinicians prefer a salpingectomy because they find this intervention easier to perform and more quickly done than a salpingostomy.

Although short term costs are lower for salpingectomy, in the long term it might be more costly because of the associated fertility problems in case of unfulfilled child wish [17]. As earlier demonstrated in a threshold analysis, based on retrospective data, salpingostomy would already be more cost-effective than salpingectomy followed by three cycles of IVF when the increase in spontaneous IUP exceeded a mere 2%, which corresponds with a FRR of 1.05 [18]. On the other hand, if salpingostomy would not be better than salpingectomy, the number of prevented cases of persistent trophoblast and repeat EP might very well tip the balance, and lead to potential savings in the other direction.

This randomised controlled trial aims to provide the final evidence in the trade off between salpingostomy and salpingectomy for tubal EP in view of the remaining uncertainties of both interventions and will offer guidance to clinicians in their decision making.

## Competing interests

The authors declare that they have no competing interests.

## Authors' contributions

FM drafted the paper and has responsibility for the logistical aspects of the trial. AS, DJ, TY are responsible for the trial in Sweden, United Kingdom and the USA, respectively, and commented on the draft paper. WMA, FvdV and BWMM contributed to the development of the protocol and commented on the draft paper. PJH was responsible for the development of the protocol, had overall responsibility for the trial, applied for a grant and commented on the draft paper. All authors read and approved the final paper.

## Pre-publication history

The pre-publication history for this paper can be accessed here:


